# Key Variables for Effective eHealth Designs for Individuals With and Without Mental Health Disorders: 2^12-4 Fractional Factorial Experiment

**DOI:** 10.2196/23137

**Published:** 2021-03-24

**Authors:** Armando J Rotondi, Jonathan Grady, Barbara H Hanusa, Gretchen L Haas, Michael R Spring, Kaleab Z Abebe, James Luther, John Gurklis

**Affiliations:** 1 Mental Illness Research, Education and Clinical Center (MIRECC) VA Pittsburgh Health Care System Department of Veterans Affairs Pittsburgh, PA United States; 2 Center for Health Equity Research and Promotion (CHERP) VA Pittsburgh Health Care System Department of Veterans Affairs Pittsburgh, PA United States; 3 University of Pittsburgh Center for Behavioral Health, Media, and Technology University of Pittsburgh Pittsburgh, PA United States; 4 Computer Science Thomas College Waterville, ME United States; 5 Department of Psychiatry School of Medicine University of Pittsburgh Pittsburgh, PA United States; 6 Department of Information Sciences and Technology School of Information Sciences University of Pittsburgh Pittsburgh, PA United States; 7 Center for Research on Health Care Data Center School of Medicine University of Pittsburgh Pittsburgh, PA United States; 8 Behavioral Health VA Pittsburgh Health Care System Department of Veterans Affairs Pittsburgh, PA United States

**Keywords:** schizophrenia, severe mental illness, eHealth, eHealth design, website, usability, website design, website usability, fractional factorial design

## Abstract

**Background:**

eHealth applications not only offer the potential to increase service convenience and responsiveness but also expand the ability to tailor services to improve relevance, engagement, and use. To achieve these goals, it is critical that the designs are intuitive. Limited research exists on designs that work for those with a severe mental illness (SMI), many of whom have difficulty traveling for treatments, reject or infrequently seek treatment, and tend to discontinue treatments for significant periods.

**Objective:**

This study aims to evaluate the influence of 12 design variables (eg, navigational depth, reading level, and use of navigational lists) on the usability of eHealth application websites for those with and without SMI.

**Methods:**

A 2^12-4^ fractional factorial experiment was used to specify the designs of 256 eHealth websites. This approach systematically varied the 12 design variables. The final destination contents of all websites were identical, and only the designs of the navigational pages varied. The 12 design elements were manipulated systematically to allow the assessment of combinations of design elements rather than only one element at a time. Of the 256 websites, participants (n=222) sought the same information on 8 randomly selected websites. Mixed effect regressions, which accounted for the dependency of the 8 observations within participants, were used to test for main effects and interactions on the ability and time to find information. Classification and regression tree analyses were used to identify effects among the 12 variables on participants’ abilities to locate information, for the sample overall and each of the 3 diagnostic groups of participants (schizophrenia spectrum disorder [SSD], other mental illnesses, and no mental illness).

**Results:**

The best and worst designs were identified for each of these 4 groups. The depth of a website’s navigation, that is, the number of screens users needed to navigate to find the desired content, had the greatest influence on usability (ability to find information) and efficiency (time to find information). The worst performing designs for those with SSD had a 9% success rate, and the best had a 51% success rate: the navigational designs made a 42% difference in usability. For the group with other mental illnesses, the design made a 50% difference, and for those with no mental illness, a 55% difference was observed. The designs with the highest usability had several key design similarities, as did those with the poorest usability.

**Conclusions:**

It is possible to identify evidence-based strategies for designing eHealth applications that result in significantly better performance. These improvements in design benefit all users. For those with SSD or other SMIs, there are designs that are highly effective. Both the best and worst designs have key similarities but vary in some characteristics.

## Introduction

### Background

Schizophrenia spectrum disorder (SSD) and other severe mental illnesses (SMIs) are commonly chronic, require ongoing treatment and support to obtain optimal health, and reduce the occurrence of symptom relapses and hospitalizations [1]. Unfortunately, treatments may not be sought, disengagement from mental health services is common, and individuals’ impressions of these services are not always favorable. Approximately 50% of those with a SMI do not seek mental health treatment in any given year [2], and 75%-85% discontinue their antipsychotic medications for a significant period during any 2-year period [3]. In addition to medications, psychosocial services (eg, case management, family psychoeducation, and cognitive behavioral therapy) are key components of evidence-based treatment for those with an SMI because they improve well-being over and above medications alone [1,4]. However, for many, there is a lack of availability of evidence-based and effective psychosocial services [5]. In addition, and similar to medication use, noninitiation, poor adherence, and discontinuation are common [5]. Unfortunately, not receiving psychosocial services and/or medications is associated with poorer outcomes, including increased risk of relapse, hospitalization, suicide, and homelessness [1,6]. In one study, 49% of those with an SMI did not seek treatment in the past year, yet said they had a problem needing professional help [2]. When individuals with a mental illness were asked why they disengaged from treatment, they cited not being listened to, unsympathetic providers, lack of participation in decisions, and being dissatisfied with services [7]. Services that can improve availability, can meet service users where they are in terms of their needs and priorities, can be tailored to their individual preferences, can be integrated with their lifestyles, and can lead to improved patient-focused outcomes are needed.

The use of eHealth technologies is an innovative approach for creating delivery models that can achieve these goals. They have the potential to support services that are more widely accessible, convenient, personalized, and able to adapt to the needs of individual users. Unfortunately, although there are many examples of success [8,9], the use of eHealth technologies has not always been successful. Dr Eysenbach advanced the *Law of Attrition (Or: Why Do eHealth Users Discontinue Usage?)* [10]. In one of his cited examples, only 1% (12/1161) of enrollees completed a 12-week panic disorder web program. In another example, an *open* evaluation of MoodGym, a proven-to-be-effective web-based intervention for depression, 99.5% of participants discontinued before completing the 5 modules. Although initial interest on the part of participants for the interventions was high, enthusiasm was lost for several reasons, including that the websites were too complex, not intuitive, and had poor usability. The Law of Attrition by Dr Eysenbach highlights the critical need to design highly usable, intuitive eHealth interventions that are accessible even to those with low technical and reading skills. Unfortunately, for those with SMIs, who may have special cognitive needs, when eHealth applications are created using design models for the general public, the designs are ineffective, often to the point of being unusable [11,12].

### Dearth of Empirical Basis for Designing eHealth Services

Due to insufficient research, there is a critical gap in the knowledge base needed to design eHealth technologies for individuals with SMI and those with cognitive impairments, special cognitive needs, and/or low technology skills [13]. Some sources for guidance do exist. A number of design guidelines are inclusive of conditions relevant to SMI [13,14]. Commonly, guideline recommendations are meant to be general enough to apply across several health conditions and illnesses. As a consequence, the recommendations can vary among guidelines, even for a given population or group. For example, a synthesis of recommendations from 20 existing guidelines [14], all of which addressed individuals with conditions that included cognitive impairments, identified only 3 recommendations that were endorsed by more than 50% of the guidelines. These recommendations, starting with the most frequently endorsed, were to use pictures, icons, and symbols with the text; use clear and simple text; and use consistent navigation and design on every page. Only the latter 2 recommendations are consistent with the empirical base with SMI [11,12,15-18], although the specifics of how to achieve these were not described. Another common limitation is that because a guideline may be created to cover a broad range of conditions, the recommendations may be more appropriate for some than others. In the previous example, the first and most commonly endorsed recommendation has been found to be a poor design feature for those with SSD and others with SMI [19,20].

There have also been recommendations based on empirical usability studies. There is consistency in the findings for some of these recommendations and inconsistency with others. Currently, consistent recommendations include the use of *large navigation buttons* [11,15,17,18]; *text at a low reading level*, preferably fourth to fifth grade [11,12,15,18,20]; *a shallow navigational hierarchy* [11,12,16-18,20]; *explicit or concrete wording* of hyperlinks, labels, and headings [11,12,17]; *pop-up menus* that appear via cursor hovering, to aid navigation [11,20]; and the *language of intended users* [12,16].

Recommendations that have been inconsistent include the font size, of which some have found it is better to increase the size over standard and use larger font [15,18,21], whereas others have found using smaller font works best [20]. In this study, we report that the effect of font size can be dependent on the design environment and on whether the desired goal is to improve the ability to find information or to reduce the time it takes to find information, to use designs that are more exciting, and to be able to grab users’ attention [22], whereas others have found the opposite, that plain designs with little distracting and superfluous content, images, or displays are best [19,20]. What is most needed to improve eHealth designs for those with SMI, as well as others, is evidence-based recommendations derived from empirical usability investigations that collect valid quantitative performance data. As the empirical foundations expand, additional recommendations will emerge, and current inconsistencies will be resolved.

### Objective

We have conducted a research program focused on identifying the design needs of individuals with SMI [12,17,20,23], as have others [11,15,18,24]. On the basis of this evidence-base, we selected a set of 12 eHealth screen design variables with the potential to have the largest effects on usability for individuals with SMI. The aim of this study is to examine the relative influence of these 12 variables on the ability of users to navigate websites. To accomplish this, we algorithmically specified the designs and then created 256 websites for testing. These websites systematically varied the 12 design variables via a 2^12-4^ fractional factorial design [25], enabling an equivalent representation of the 12 design variables in testing combinations of the design. The final destination pages of all websites were identical; only the designs of the navigational screens and pathways were different. For analyses, participants were divided into 3 diagnostic groups: those with a SSD diagnosis, those with any other mental health diagnosis, and those with no mental health diagnosis.

## Methods

### Participant Recruitment and Training

Participants were recruited via convenience sampling from the Department of Veterans Affairs (VA) Pittsburgh Health Care System and non-VA community mental health outpatient treatment centers. The enrollment criteria were as follows: aged at least 18 years, physical ability to read the screen of a computer and use a mouse, and ability to read at a fifth-grade level. There were no requirements for prior computer, mouse, internet, or website use. This study was approved by the VA Pittsburgh Healthcare System Institutional Review Board.

To screen for reading ability, participants completed the reading subtest of the Wide Range Achievement Test. Data were collected over 3 separate sessions. In the first session, demographic data, questions about past computer use, and the Structured Clinical Interview for the Diagnostic and Statistical Manual of Mental Disorders-IV (DSM-IV) were administered to all participants to assist in determining the presence of DSM-IV Axis I mental disorders [26]. One of the questions was to self-rate one’s level of computer understanding using a 5-point scale. In the analyses, this was used as a proxy for how savvy participants were with technology. To simplify the analyses, this was collapsed into 3 levels of self-rated expertise: responses 1 and 2 represented *low*, 3 was *moderate*, and 4 and 5 were *high*. In the second session, a neurocognitive functioning measurement battery was administered to evaluate basic cognitive abilities. In the third session, participants were tasked to find information on 8 different websites. To ensure that all participants had the basic skills needed to navigate the websites, each was taken through a brief tutorial (covering topics such as mouse use, hyperlink text, pop-up menus, and page scrolling) [12]. All individuals who met the eligibility criteria were able to master these basic skills. Following this, the performance of the tested websites was evaluated.

### Design of the Websites and Selection of the 12 Variables

In total, 12 interface design variables (Table 1), each at 2 levels, were used to algorithmically generate a full factorial design of 2^12^ (ie, 4096) website designs. From these 4096 website designs, we identified an algorithmically derived *fractional* set of 256 websites to create. That is, the design of each of the 256 websites was specified via a sequential 2^12-4^ fractional factorial [25], which is a common industrial engineering experimental design. We have been conducting a research program focused on identifying the eHealth design needs of individuals with SMI and have included in this program a focus on SSD. This research program created an eHealth design model for those with SMI, termed the flat explicit design model (FEDM) [12], which was initially composed of 6 design variables. As the program progressed, the FEDM grew to 18 design variables [17]. The 12 variables for this study were chosen to be the most influential design variables, based on a literature and internet review of design recommendations and our experiences creating and evaluating eHealth applications for those with SMI [27], and to be the most important from the 18 variables of the FEDM. The reading level of website content was assessed using the Flesch-Kinkaid reading scale (as assessed by Microsoft Word) [28]. The contents (ie, topics and final destination articles) were taken from a previously created web-based intervention [27] and were identical in all websites; only the navigational designs of websites differed.

**Table 1:** The 12 website design variables.

Wording used for design feature	Design feature	Levels of dimensions in the factorial design	
		Low	High
Navigational depth	Number of pages one needs to navigate to get from the home page to a desired piece of information.	≤3	≥5
Number of hyperlinks on a page	Each hyperlink is counted as one link. They may be embedded in standard text (ie, nonhyperlinked text) or stand alone, buttons (images, icons, and logos) or tool bars, and pull-down/pop-up menus. This variable is the total number of hyperlinks on a page.	≤7	≥14
Pop-up menu	Use of a pop-up menu to display the hyperlinks on the page.	0	1
Reading level	The grade level of links, labels, and text.	≤7	≥9
Words per page	Number of words on a page.	<100	≥200
Screen length	Screen length of navigation pages (paging vs scrolling)	≤1	>1
Number of distinct navigation areas	The number of separate areas on a page where users will find hyperlinks that can navigate the site.	≤2	≥4
Font size of body text	The size of the text used in the website	10 point	13 point
Number of words per hyperlink	The number of words used in the hyperlinks to the application’s contents.	≤3	≥6
Number of nonhyperlink graphic elements on a page	This includes images, pictures, graphics (eg, color bars), and figures on a page that are not hyperlinks. They are for *decoration* or illustration only.	0	≥3
Constant navigational toolbar	Navigational tool bars that are in the same area across all pages on a website.	0	1
Number of topic areas on a page	A topic is an area devoted to one subject, purpose, or theme. For example, it could be a welcome paragraph; a list of links to main topics in the website (with or without introductory text); a list of links to news about the latest research on a topic; or a place to enter data, such as a search engine box.	≤4	≥6

### Website Design and Evaluation Procedures

Each subject was asked to find information on 6 specified topics, on each of the 8 websites. Our previous experience indicated that this level of effort would not overly tax anyone who met our selection criteria. Given that we did not want fatigue to influence the results during testing, we chose a relatively conservative number. Example tasks were to find information on *what causes schizophrenia*, *how schizophrenia is treated*, and *the side effects of medications used to treat schizophrenia*. Two steps were taken to eliminate a *learning* effect influencing the results across the sample of participants. The order of the 6 tasks and the order of the 8 websites varied from individual to individual. The task order for each website was assigned using randomly permuted blocks of size 6. To vary the order of testing of the websites across participants, the order was assigned using randomly permuted blocks of 8. Testing occurred in a research office. If a subject selected an item as his or her choice but the item was not the target, he or she was informed and instructed to continue searching for the item until the allotted task time had expired (3 minutes). Tasks were timed unobtrusively using a browser plugin that we had developed. When the time allotted to complete a task expired, the computer screen automatically went blank, and the next task was initiated. Recording of the website usability testing was accomplished using applications that we developed to operate with the Firefox internet browser. To minimize testing anxiety, each participant was read a script explaining that the procedures were to evaluate the websites and not the participant and that there were no right or wrong answers.

Participants’ abilities to locate the requested information (ie, design effectiveness) and time to accomplish tasks (ie, design efficiency) were analyzed to identify the design variables and combinations of variables that created more or less usable websites (ie, facilitate or inhibit use). The maximum time allowed to look for information was 180 seconds per task. To eliminate the over dispersion of times to success, the time data were analyzed using the natural logarithms of seconds to find.

### Statistical Analyses

Comparisons of baseline demographics across the 3 mental health groups were completed with analyses of variance for continuous measures and chi-square for categorical measures, with exact tests as needed for small samples. Percentages of success and times to success were averaged over each website. Regression tree analyses were used to identify patterns of the 12 dimensions that led to more or fewer successes at finding the required information or more or less time to successfully find the information.

Comparisons of performance across each of the 12 website dimensions were performed first on the entire sample (n=222 participants for ability to find information and n=218 for time to find information because 4 participants did not find any of the targeted information) and then for each of the 3 diagnostic groups separately, using classification and regression trees (CART) for success (CART Salford Predictive Modeler v8.2) [29]. The main effects were tested for all dimensions, and the interactions indicated as potentially significant by CART were tested with mixed effect regression models. Nesting of website performance within an individual was included in the regressions.

Regression tree procedures identify, among the predictor variables, the most efficient variable for splitting the observations into 2 groups. For example, it selected the 1 website dimension that best split higher performing websites (in terms of number of tasks completed successfully) versus lower performing websites. After the first split, each branch (descendent group) was split by the most efficient predictor variable within that diagnostic group. This created another level of branching. The amount of branching that occurs can be determined by specifying the number of observations in a descendent group that stops the branching or the number of branches desired. In this study, the tree was based on the percentage of tasks within a website that were completed correctly, and the branching was stopped with <12 person observations per website. As regression tree analyses do not account for multiple observations per individual subject, mixed effect regressions that can account for multiple assessments evaluated by the same individual were performed. The results of the regression tree analyses were used to identify possible interactions that would be tested in a multilevel model. Initial regression analyses included the 12 dimensions and interactions identified in the regression trees as well as the diagnostic variables of the 3 groups. Backward stepped regressions were used to remove the nonsignificant main effects and interactions. These models also indicated that the differences between the 2 non-SSD groups were not significant at the main effect or interaction effect level, and these 2 groups were combined and served as the baseline diagnostic group.

## Results

### Sample Description

**Figure 1:**
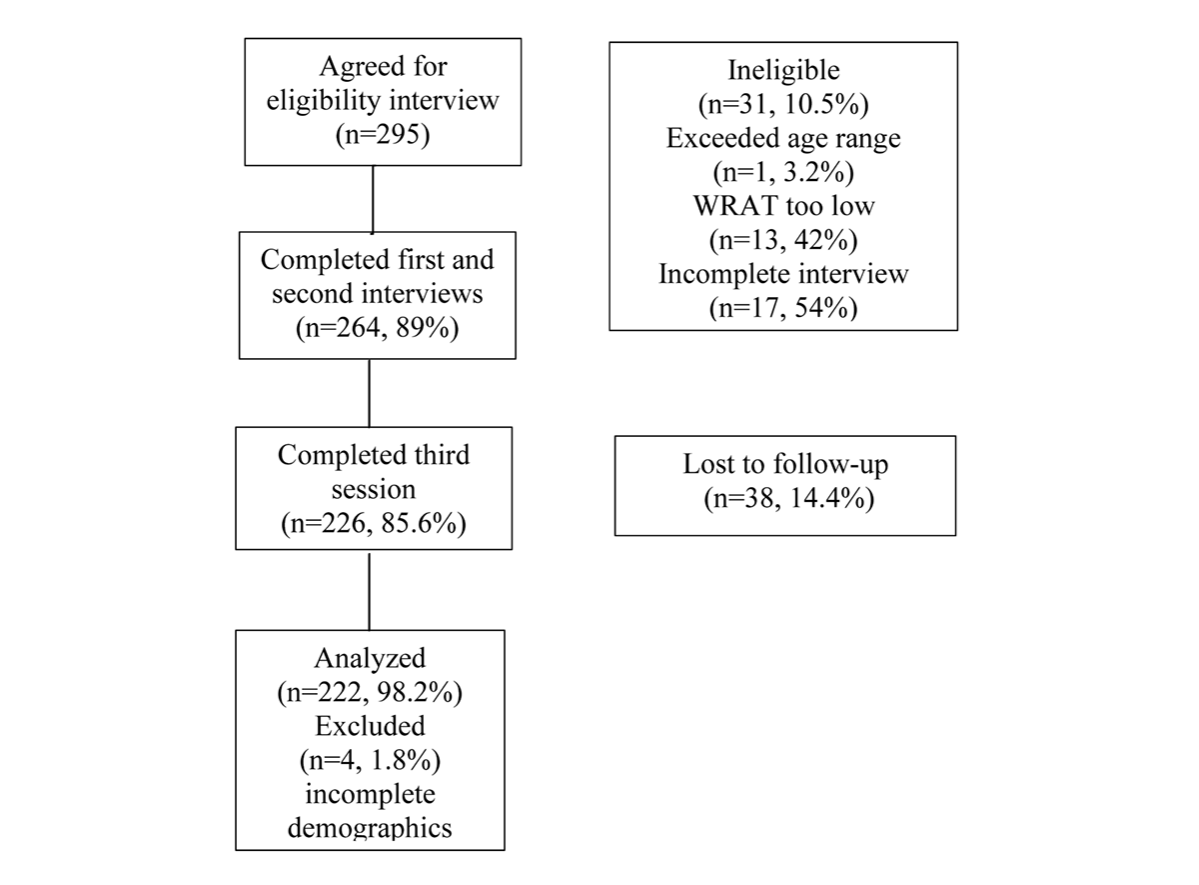
Consolidated Standards of Reporting Trials chart. WRAT: Wide-Range Achievement Test.

A total of 295 participants met the eligibility criteria (Figure 1). Of these 295 participants, 264 (89.5%) completed the first and second data collection sessions for the study, and 226 (85.6%) completed the third session where the performance of the websites was assessed. Of the 226 participants, 4 were missing key demographic and/or computer experience information, resulting in 222 (75.2%) of the original 295 participants for this report. On the basis of mental health diagnoses, the 222 participants were placed in 1 of 3 diagnostic groups: no mental illness (83/222, 37.4%), mental illness without evidence of psychotic features (eg, depression and anxiety; 60/222, 27.0%), and SSD (79/222, 35.6%).

The 222 participants ranged in age from 23 to 75 years (mean 52.2, SD 9.2), and African Americans comprised 49.5% (110/222) of the sample (Table 2). Those with SSD, when compared with the other 2 groups, were more likely to be male, less likely to be employed, and more likely to be retired or disabled. In terms of technology, individuals with SSD were less likely to have a computer available at home, more likely to self-assess as having little or no computer understanding, and less likely to self-assess as having high computer understanding.

**Table 2:** Demographic and clinical characteristics split by diagnostic group (N=222).

Demographic variable		SSD^a^ (n=83)		Mental illness other than SSD (n=60)		No current mental health diagnoses (n=79)			Comparison of the 3 diagnostic groups (*P* value)	
Age (years)^b^, mean (SD); range		53.1 (8.7); 29-71		52.1 (8.6); 23-72		51.6 (10.2); 23-75			.58	
**Gender, n (%)^c^**								.02		
	Male		69 (84)		45 (75)		50 (63)			
	Female		14 (16)		15 (25)		29 (37)			
**Race, n (%)^d^**								.11		
	White		46 (58)		24 (40)		49 (51)			
	African American		35 (42)		36 (60)		39 (39)			
**Education level, n (%)^e^**								.14		
	Less than or equal to high school graduate		39 (47)		16 (27)		31 (39)			
	Post high school training		35 (42)		33 (55)		34 (43)			
	College degree or more		19 (9)		11 (18)		14 (18)			
**Employment status, n (%)^f^**								<.001		
	Full or part time		15 (18)		23 (38)		39 (49)			
	Retired/disability only		68 (82)		37 (62)		40 (51)			
**Self-reported computer understanding, n (%)^g^**								.06		
	None/little		31 (38)		11 (18)		19 (24)			
	Some		28 (34)		22 (37)		25 (32)			
	Good/complete		23 (28)		27 (44)		34 (44)			
Computers available at home, n (%)^h^		28 (34)		34 (57)		44 (56)			.006	

^a^SSD: schizophrenia spectrum disorder.

^b^*F*_2221_=0.55.

^c^χ^2^_2_=8.3.

^d^χ^2^_4_=4.4.

^e^χ^2^_4_=6.9.

^f^χ^2^_2_=18.0.

^g^χ^2^_4_=9.0.

^h^χ^2^_4_=10.3.

### Website Usability

#### All Participants Pooled

When all participants for the 256 websites were pooled, the average rate of success at finding information was 33.2%. The mean time to correctly find information was 49.0 seconds (median 40, range 7-180). The SSD group, when separately compared with the other 2 groups (ie, mental illness other than SSD and no mental illness), had a lower success rate (*P*=.007 for each) and took longer to find information (*P*=.001 for each; Table 3). All the 256 websites that were used in this study were created specifically for the study. Consequently, no participant had any experience with the websites they were tested on and were seeing and using each website for the first time.

**Table 3:** Overall performance (N=222).

Ability to find information	SSD^a^, n=83		Mental illness other than SSD, n=60		No current mental health diagnoses, n=79		Significance
	Mean (SD)	Median	Mean (SD)	Median	Mean (SD)	Median	
Success rate at finding information per website (%)	28.6 (29.4)	16.7	37.8 (30.9)	33.3	34.5 (30.3)	33.3	Median, *P*=.007^b^
Time to find information per website (s)	59.8 (38.0)	51.1 (8-180)	44.4 (31.1)	35.3 (7.3-180)	42.8 (30.9)	35.3 (9.5-180)	Mean, *P*<.001^b^

^a^SSD: schizophrenia spectrum disorder.

^b^Significance levels based on mixed models that accounted for nesting of websites within participants.

### Which Design Variables Influence Ability to Find Information: Mixed Regression Models, All Participants

Mixed regression models were created to identify the variables with main effects and interaction effects on the ability of participants to successfully find information. One of the models (Table 4, model 1) included the 12 design dimensions (Table 2) and the 3 diagnostic groups. In this model, the higher level of each of the 6 design variables leads to worse performance: higher navigational depth (≥5), higher number of hyperlinks on a page (≥14), higher reading level (≥ninth grade), larger font size (13 points), longer than 1 screen length of contents (ie, contents that require scrolling to see all of it), and higher number of navigation areas on a page (≥4); however, the higher level of 1 design variable use of a pop-up menu to display hyperlinks on a page improved performance. Having an SSD had a negative influence on the ability to find information (although it must be noted that this occurred within the 3-minute time limit imposed on finding a given piece of information). There were no significant differences between the other 2 diagnostic groups, that is, the difference was between SSD and the other 2 groups combined. A second mixed regression model (Table 4, model 2) was developed that included the self-rated measure of participant computer understanding. With this variable in the model, SSD (vs others) was no longer a significant variable.

**Table 4:** Multivariable models of percentage of tasks completed successfully.

Variables			Coefficient	95% CI	Significance (*P* value)
**Model 1: Design dimensions and diagnostic groups**					
	**Dimensions**				
		Navigational depth	−0.318	−0.348 to −0.289	<.001
		Hyperlinks per page	−0.083	−0.108 to −0.060	<.001
		Pop-up menu	0.056	0.032-0.080	<.001
		Navigational depth×Navigational lists per page	−0.031	−0.067 to 0.003	.08
		Reading level	−0.050	−0.073 to −0.026	<.001
		Large font	−0.036	−0.059 to −0.012	.003
		Read level×large font	0.046	0.012-0.080	.008
		Words per page	−0.020	−0.044 to 0.004	.10
		Navigational depth×words per page	−0.038	−0.073 to −0.004	.03
		Screen length	−0.043	−0.066 to −0.020	<.001
		Hyperlinks per page×screen length	0.037	0.003-0.070	.03
		Navigation areas per page	−0.018	−0.035 to 0.000	.05
	**Diagnostic group**				
		SSD^a^ (vs others)	−0.059	−0.104 to −0.013	.01
		Constant	0.604	0.563-0.644	<.001
**Model 2: Design dimensions, diagnostic groups, and computer understanding**					
	**Dimensions**				
		Navigational depth	−0.318	−0.348 to −0.289	<.001
		Hyperlinks per page	−0.084	−0.108 to −0.060	<.001
		Navigational lists per page	0.056	0.031-0.080	<.001
		Navigational depth×navigational lists per page	−0.030	−0.065 to 0.004	.09
		Reading level	−0.049	−0.073 to −0.025	<.001
		Large font	−0.036	−0.059 to −0.013	.002
		Read level×large font	0.048	0.0125-0.082	.005
		Words per page	−0.021	−0.045 to 0.003	.09
		Navigational depth×words per page	−0.038	−0.072 to −0.004	.03
		Screen length	−0.044	−0.067 to −0.020	<.001
		Hyperlinks per page×screen length	0.038	0.005 to 0.071	.03
		Navigation areas per page	−0.017	−0.035 to 0.000	.05
	**Diagnostic group**				
		SSD (vs others)	−0.039	−0.110 to 0.032	.28
	**Self-reported level of computer understanding**				
		None/minimal (vs high)	−0.260	−0.328 to −0.192	<.001
		Some (vs high)	−0.066	−0.112 to −0.019	.01
		None/minimal understanding×navigational depth	0.089	0.467 to 0.130	<.001
		Some understanding×navigational depth	0.024	−0.016 to 0.064	.24
		Constant	0.687	0.640 to 0.735	<.001

^a^SSD: schizophrenia spectrum disorder.

#### Design Variables That Influence Time to Find Information: All Participants

Mixed regression models were created to identify the variables with main effects and interaction effects on the time it took participants to correctly find information. One of the models included the 12 design dimensions and 3 diagnostic groups (Table 5, model 1). Five of the main effects were the same variables as for the ability to find information (Table 4, model 1), and 4 of these at the higher level also had a negative effect on the time it took to find information. These variables had a negative effect on the time to find information: higher navigational depth, higher number of hyperlinks on a page, higher reading level, and pages longer than 1 screen length. The fifth variable, larger font, positively influenced time to find information, that is, reduced the amount of time needed. In addition, the following 3 variables had a negative effect at their higher levels: higher number of words per page (≥200), presence of a tool bar, and more words per hyperlink (≥6). In addition, SSD (vs others) was the only diagnostic group that entered the model, and it had a negative effect.

A second mixed regression model was developed (Table 5, model 2), which, in addition to the above variables, included the self-rated measure of participants’ computer understanding. This entered the model as a significant variable. All of the other variables and 2-way interactions from model 1 remained in the model, including SSD.

**Table 5:** Multivariable models of the time to correctly find information.

Variables			Coefficient	95% CI	Significance (*P* value)
**Model 1: Design dimensions and diagnostic groups**					
	**Dimensions**				
		Navigational depth	0.418	0.358 to 0.479	<.001
		Hyperlinks per page	0.300	0.205 to 0.396	<.001
		Reading level	0.208	0.131 to 0.285	<.001
		Reading level×hyperlinks per page	−0.147	−0.263 to −0.029	.01
		Navigational lists per page	0.057	−0.019 to 0.134	.14
		Words per page	0.092	0.017 to 0.167	.02
		Words per page×hyperlinks per page	−0.108	−0.219 to 0.003	.06
		Toolbar	0.146	0.052 to 0.240	.002
		Navigational lists per page ×toolbar	−0.138	−0.247 to −0.027	.01
		Large font	−0.070	−0.122 to −0.017	.009
		Screen length	0.054	0.002 to 0.106	.04
		Words per hyperlink	0.092	0.013 to 0.170	.02
		Words per hyperlink×toolbar	−0.101	−0.208 to 0.005	.06
	**Diagnostic group**				
		SSD^a^ (vs others)	0.347	0.221 to 0.473	<.001
		Constant	3.216	3.090 to 3.342	<.001
**Model 2: Design dimensions, diagnostic groups, and computer understanding**					
	**Dimensions**				
		Navigational depth	0.411	0.371 to 0.492	<.001
		Hyperlinks per page	0.303	0.208 to 0.398	<.001
		Reading level	0.200	0.123 to 0.276	<.001
		Hyperlinks per page ×read level	−0.147	−0.264 to −0.031	.01
		Words per page	0.096	0.021 to 0.171	.01
		Hyperlinks per page×words per page	−0.112	−0.022 to −0.001	.048
		Navigational lists per page	0.052	−0.025 to 0.129	.18
		Toolbar	0.150	0.055 to 0.245	.002
		Navigational lists per page×toolbar	−0.141	−0.250 to −0.030	.01
		Large font	−0.073	−0.126 to 0.061	.006
		Screen length	0.130	0.050 to 0.210	.002
		Words per hyperlink	0.097	0.019 to 0.175	.02
		Words per hyperlink×toolbar	−0.104	−0.211 to 0.003	.06
	**Diagnostic group**				
		SSD (vs others)	0.256	0.140 to 0.372	<.001
	**Self-reported level of computer understanding**				
		None/minimal (vs high)	0.555	0.399 to 0.712	<.001
		Some (vs high)	0.257	0.116 to 0.397	<.001
		**Screen length×self-reported level of computer understanding**			
		None/minimal	−0.103	−0.242 to 0.034	.14
		Some	−0.135	−0.255 to −0.016	.03
		Constant	2.939	2.804 to 3.074	<.001

^a^SSD: schizophrenia spectrum disorder.

### Designs That Increased or Reduced Usability in Terms of Ability to Find Information: CART Data Analysis Using All Participants and All Websites

**Figure 2:**
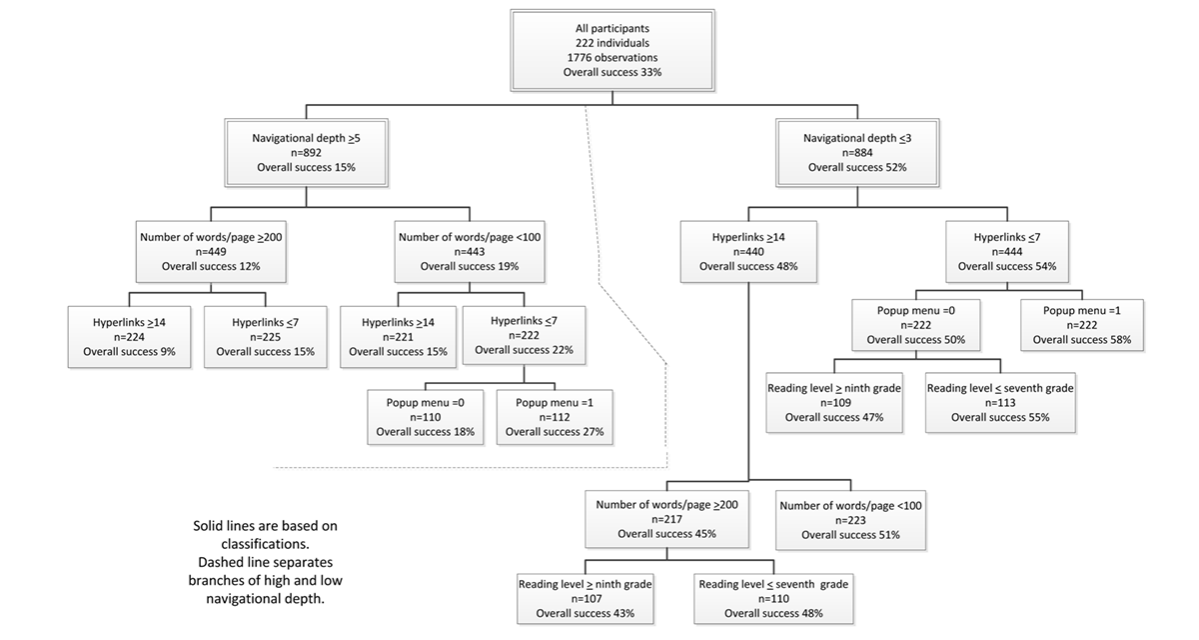
Classification and regression trees analyses of all participants.

The variable with the greatest influence on participants’ abilities to successfully find information was navigational depth (Figure 2). For designs with deep navigation (ie, a navigational depth of ≥5 levels or screens), the average percent success was only 15%. For this subset, if in addition they had ≥200 words per page, and ≥14 hyperlinks per page the success rate decreased to 9%. This was the worst performing combination of the design elements tested in this category. The performance of designs with high navigational depth could be improved to a still exceedingly poor 27% success rate by using <100 words per page, ≤7 hyperlinks per page, and a pop-up menu to present the hyperlinks on a page.

For websites designed with shallow navigation (ie, navigational depth ≤3 levels), the average success rate over all such designs and all participants was 52% (Figure 2). In addition, if they had ≤7 hyperlinks per page (54%) and used a pop-up menu to present the hyperlinks, the average success rate increased to 58%.

Of the designs with shallow navigational depth, the worst performing design combination had ≥14 hyperlinks per page (48%), ≥200 words per page (45%), and text with a reading level that was greater than or equal to ninth grade (43%).

### Participants With SSD

#### Which Designs Make Usability Worse?

**Figure 3:**
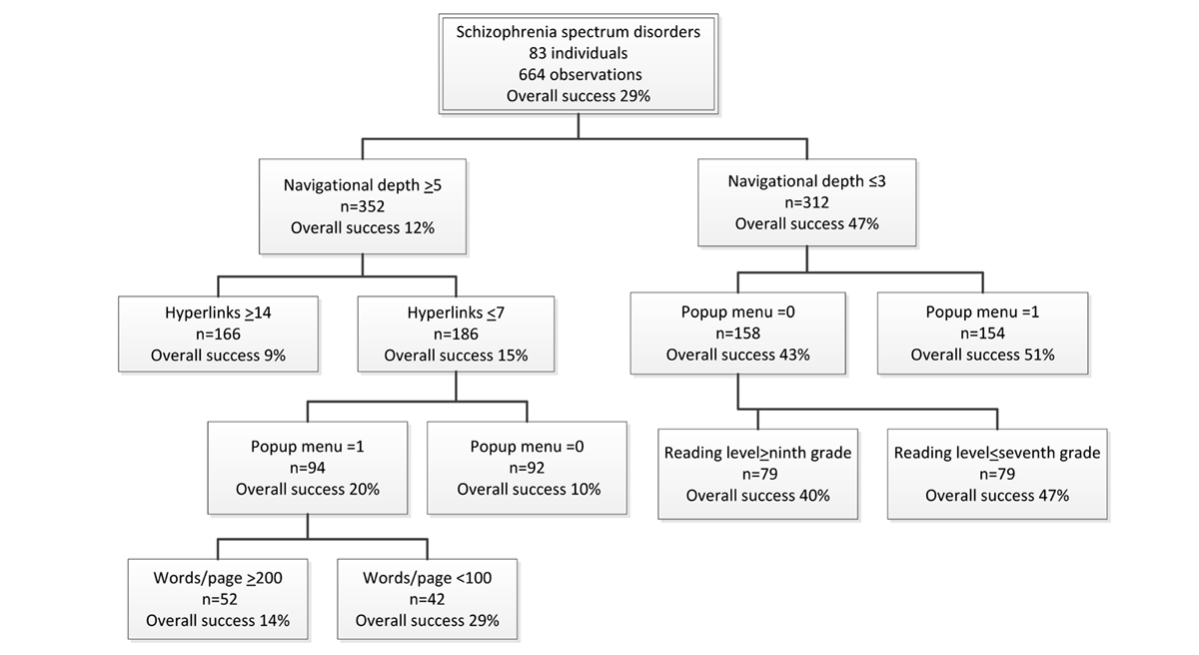
Classification and regression trees analyses participants with schizophrenia spectrum disorder.

For individuals with SSD (n=83), the average success rate for all tested websites was relatively low (29%; Figure 3). The average success rate for all designs with deep navigational depth was only 12%. For these designs, if they had ≥14 hyperlinks per page, it fell to 9%. This was the worst performing design for this group of participants. Furthermore, other designs were apparently worse, but there were not enough subjects for the differences to be statistically significant.

The highest performing designs of those with deeper navigational depth had a success rate of only 29%. This improvement was achieved by having ≤7 hyperlinks per page (15%), using a pop-up menu to present the hyperlinks on each page (20%), and having <100 words per page. Using these design elements in an overall poor design helped to improve performance but only modestly, and these designs were still quite poor.

#### Which Designs Make Usability Better?

The average success rate for designs with shallow navigational depth was 47%. The best design within this set, with a success rate of 51%, was achieved by using a pop-up menu to present the hyperlinks. For websites with shallow navigation, the worst performing designs had a success rate of 40%. This occurred for websites with no pop-up menu and greater than or equal to ninth-grade reading level.

### Participants With a Mental Illness Other Than SSD

#### Which Designs Make Usability Worse?

**Figure 4:**
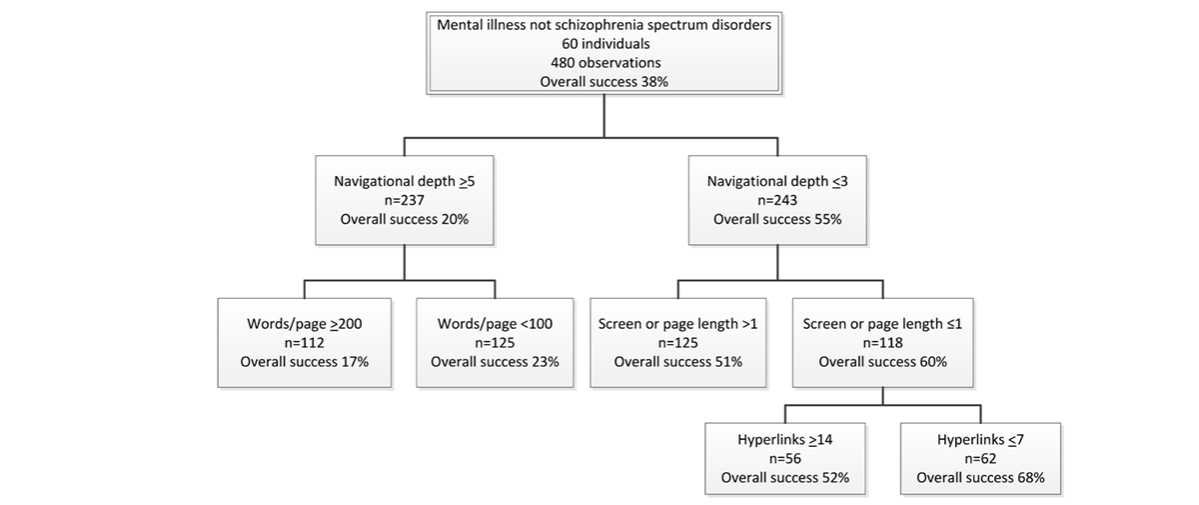
Classification and regression trees analyses of participants with a mental illness other than schizophrenia spectrum disorder.

The average success rate for all tested designs with individuals with a mental illness other than SSD was 38% (Figure 4), and for all designs with deeper navigational depth, it was 20%. Performance was reduced to 17% for these latter designs if they also had ≥200 words per page. If the deeper navigational depth designs instead had <100 words per page, the success rate increased to 23%, which was the best performing deep navigational depth design for this group.

#### Which Designs Make Usability Better?

For all shallow navigational depth designs, the average success rate was 55%. Performance increased to 68% in designs that had page length ≤1 screen long (60%), that is, did not require scrolling to see all of the contents, and that had ≤7 hyperlinks per page. For websites with shallow navigation, the worst performing designs had a success rate of 51%. This occurred for designs where the page length was greater than one screen in length.

### Participants With No Mental Illness

#### Which Designs Make Usability Worse?

**Figure 5:**
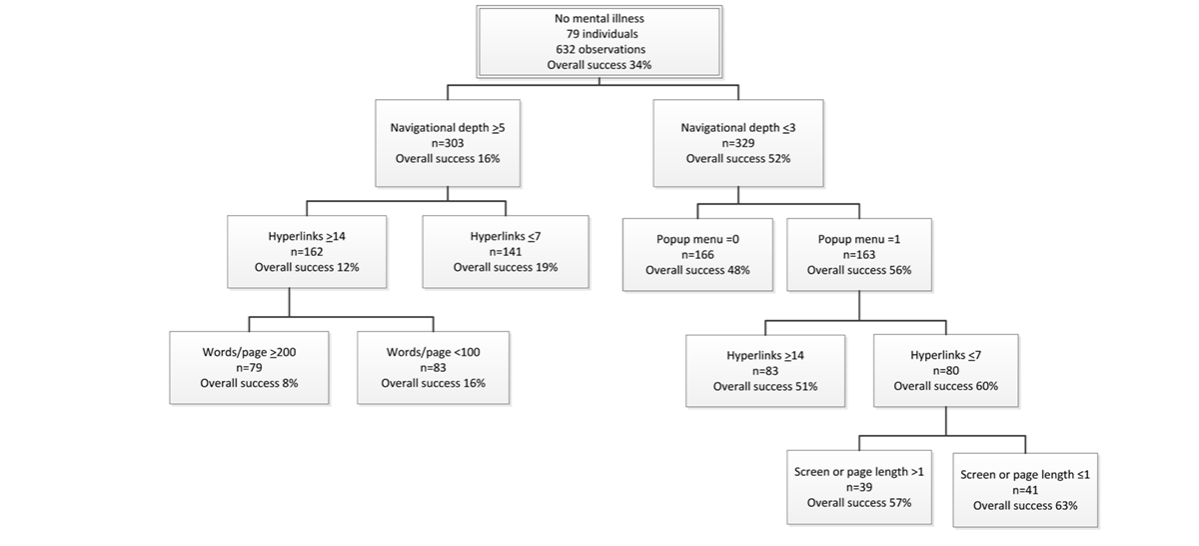
Classification and regression trees analyses of participants with no mental illness.

For participants with no mental illness, the average success rate for all tested designs was 34% (Figure 5). For the deep navigational depth designs, the average success rate dropped to 16%. The performance dropped further to a low of 8% for websites having ≥14 hyperlinks per page (12%) and ≥200 words per page. Within the set of websites that had a deep navigational hierarchy, the best performing were those with ≤7 hyperlinks per page, which achieved an average success rate of only 19%.

#### Which Designs Make Usability Better?

The websites with shallow navigational depth (ie, ≤3 levels) had an average success rate of 52%. This is more than 3 (3.25) times the average success rate of designs with deep navigation (≥5 levels). The performance increased to 63%, if websites used a pop-up menu to present the hyperlinks (56%), had ≤7 hyperlinks per page (60%), and displayed information on ≤1 screen length (ie, no scrolling was used). The worst performing designs with a shallow navigational depth had a success rate of 48%. This occurred for websites with no pop-up menu.

### Best and Worst Design Variables

Several design variables were commonly found in the best performing designs (Table 6) for each of the 4 participant groups (ie, SSD, mental illnesses other than SSD, no mental illness, and all participants); however, only shallow navigational depth was present in all 4 of these highest performing website designs. Design elements that always had a positive effect on usability were shallow navigational depth, presenting hyperlinks via a pop-up menu, ≤7 hyperlinks per page, and presenting the contents using a page length ≤1 screen long.

There were also several design elements that were commonly found on the worst performing website for each of the 4 participant groups (Table 6). High navigational depth was the only variable present in all 4 of these worst performing designs. The design elements that when present always had a detrimental effect on usability were high navigational depth, high words per page (≥200), ≥14 hyperlinks per page, page length >1 screen long, and high reading level (≥ninth grade).

**Table 6:** Best and worst performing designs and design variables for finding information.

Participant group			Design elements
**The best performing websites had these design elements**			
	Diagnostic group		Dimensions (overall success rate for the design)
	SSD^a^		Low navigational depth (≤3 levels) and pop-up menu (79/154, 51%)
	Mental illness other than SSD		Low navigational depth (≤3 levels), page length ≤1 screen long, and ≤7 hyperlinks per page (42/62, 68%)
	No mental illness		Low navigational depth (≤3 levels), pop-up menu, hyperlinks ≤7 per page, and page length ≤1 screen long (26/41, 63%)
	All participants		Low navigational depth (≤3 levels), ≤7 hyperlinks per page, and pop-up menu (129/222, 58%)
**The worst performing websites had these design elements**			
	SSD		High navigational depth (≥5 levels) and ≥14 hyperlinks per page (15/166, 9%)
	Mental illness other than SSD		High navigational depth (≥5 levels) and ≥200 words per page (19/112, 17%)
	No mental illness		High navigational depth (≥5 levels), ≥14 hyperlinks, and ≥200 words per page (6/79, 8%)
	All participants		High navigational depth (≥5 levels), ≥200 words per page, and ≥14 hyperlinks (20/224, 9%)
**The worst performing websites with low navigational depth (≤3 levels) had these design elements**			
	SSD		No pop-up menu and high reading level greater than or equal to ninth grade (32/79, 40%)
	Mental illness other than SSD		Page length >1 screen long (64/125, 51%)
	No mental illness		No pop-up menu (80/166, 48%)
	All participants		≥14 hyperlinks per page, ≥200 words per page, and high reading level greater than or equal to ninth grade (46/107, 43%)
**The best performing websites with high navigational depth (**≥**5 levels) had these design elements**			
	SSD		≤7 hyperlinks per page, pop-up menu and <100 words per page (12/42, 29%)
	Mental illness other than SSD		<100 words per page (29/125, 23%)
	No mental illness		≤7 hyperlinks per page (27/141, 19%)
	All participants		<100 words per page, ≤7 hyperlinks per page, and pop-up menu (30/112, 27%)
**Variables that, when present, always had a positive effect on performance**			
	**Variable**		Variable was also present in the best performing design for n of the 4 groups (the 3 diagnostic groups and all participants)
		Low navigational depth	4
		Pop-up menu used	3
		≤7 hyperlinks	3
		Page length ≤1 screen long	2
**Variables that, when present, always had a negative effect on performance**			
	**Variable**		Variable was also present in the worst performing design for n of the 4 groups (the 3 diagnostic groups and all participants)
		High navigational depth (≥5 levels)	4
		≥200 words per page	2
		≥14 hyperlinks per page	2
		Page length >1 screen long	0 (none)
		High reading level (≥ninth grade)	0 (none)

^a^SSD: schizophrenia spectrum disorder.

## Discussion

### Principal Findings

Overall, one of the key findings is that by varying the designs of eHealth applications in highly definable ways, it is possible to improve the effectiveness of an application for users, in terms of their success at finding information and time to find the desired information. The best designs, when compared with the worst, made a difference in the success of using a website, from 38% to 55% in the 3 diagnostic groups.

These data identify the depth of navigation, that is, the number of screens one needs to navigate through to reach the desired contents, as the most important variable for usability. This has also been identified as important by others [11,12,18,20]. When all participants’ data were pooled, the average success rate for designs with a shallow hierarchy was 3.4 times higher than the average for those with a deep hierarchy. The worst performing design with a shallow hierarchy was still 2.5 times more successful than the best design with deep navigation. This principle was held across each of the 3 diagnostic groups as well. Although we found that the design environment (ie, the screen’s overall design) influenced the effects of several design variables on usability, this was not the case for navigational depth. Its influence was invariable. The presence of other design elements influences how strong this effect is, but shallow is always better than deep, all else being equal. This indicates that a shallow navigational structure is an essential design feature for usability; therefore, creating a shallow navigational structure is an essential design challenge to address.

It should be noted that all participants were using these websites for the first time; however, cognitive limitations can restrict one’s ability to comprehend complex designs and create a mental model of an application, even after repeated use [12]. Without an accurate mental model, it is harder to become a savvy user. Consequently, it is possible that the differences that were found between the participant groups were minimal differences in what might be found if the comparisons were made after repeated website use.

Our previous research found that it was more effective to have users scroll down a screen to obtain additional information than to navigate to a new screen (ie, *scrolling* was superior to *paging*) [17,20]. This study indicates that designs that used scrolling were never the highest performing designs and tended to be inferior to those that used paging. A recent design study found that users state a clear preference for paging over scrolling [30]. The findings of this study are consistent with this preference. As we have pointed out previously, scrolling is a poor design choice, but our work indicates that, in some circumstances, it may have certain advantages to paging, if paging significantly increases the depth of navigation. Depth is the most important single variable for usability, and this finding holds for all groups. Although users prefer paging, it is more convenient, and makes it less likely that content will be missed by users, adding pages is potentially perilous to performance, particularly for less savvy users who may be more likely to become confused or lost in a deeper hierarchy. The design lesson may be that the best designs, in terms of quantitative effectiveness and user preferences, will minimize the need to scroll and will, at the same time, use a shallow navigational structure that relies on paging.

This issue is relevant to other design elements, for example, the number of hyperlinks on a page. The findings of this study were clear; the lower number of links to contents, compared with the higher number, contributed to a superior design. This study did not attempt to determine whether there might be an *optimal* number or range of navigation links on a page. The design simply compared 2 disparate levels to determine whether this might be an important design variable, and it was an important design variable. Although using a higher number of links was clearly inferior, the most important design element for users’ success was the number of screens they needed to navigate through. Therefore, in any given design, there could be a trade-off between the number of links on a screen and the navigational depth. The evidence of this study as well as past studies [17] indicate that having more links on a page, if it can significantly reduce the number of pages a user must navigate through, could be expected to be a more effective design choice, all else being equal.

For this study, we created a single question to allow participants to self-assess how savvy they felt they were with technology. We have referred to this variable as *computer understanding*. The computer understanding variable accounted for the effect of SSD in the regression model of the ability to find information. This indicates that it is not necessarily the characteristics of the illness per se, but rather familiarity and skills with technology that were a key reason for poorer performance by this group. This may be caused by less use of associated technologies. It is possible that those with SSD who have significant cognitive challenges will have greater difficulty becoming savvy and/or will benefit from having designs that specifically accommodate their cognitive needs. This study and our prior work indicate that specific design features can be helpful to those with SSD, who may have special cognitive needs, while not reducing the performance of others. A clear implication of the findings from this study is that training with eHealth technologies should improve less savvy individuals’ performance with technology. This has been observed in our prior eHealth intervention studies [27].

The findings of this study indicate that the design environment can influence the impact of a design element, at least to some extent. This indicates that there is not necessarily just one route to designing a highly usable page or screen. However, the highest performing pages did have key similarities, and there were still noticeable differences in the usability between alternative *good* designs, that is, no 2 alternative designs had the same performance. This latter point supports the premise that there are certain design principles that seem to be fundamental to creating high-performance applications.

For all participants, when the measure of computer understanding was included in the regression model for the time required to find information, SSD (vs the other 2 diagnostic groups) remained a significant variable in the model. This would suggest that there is something in addition to how savvy one may be with these technologies that influences the time it takes to find information. This was not observed in the regression model describing the ability to find information, where SSD (vs others) dropped out of the model, indicating that the relevance of computer understanding was a factor that was common to all groups combined. Deficits in processing have been found by others [31], and our own data show (manuscript in preparation) that the processing speed of those with SSD was slower than that of the other groups in the study. This might contribute to increasing the time that it takes to find information but does not influence the ability to find information.

### Limitations

There are several limitations that need to be considered when interpreting these findings. Participants had to be able to read at the fifth-grade level to enter the study. The design needs of those with lower or even much higher reading levels may be different. The sample may have been too small to detect anything but main effects or very large interactions between the variables. The experimental design was limited to an evaluation of only 12 variables. Other variables may also be, and likely are, important. As a segment of the participants had little or no familiarity with technology, all participants were taken through a brief training that showed them how to use a mouse and demonstrated all of the website navigational elements they would encounter. This likely improved the abilities of these users and their performance compared with similar individuals in the general public who would not have such training.

### Clinical Implications

One of the keys to successfully engaging consumers with eHealth treatments and services, particularly consumers with SMI and special cognitive needs, is to provide intuitive navigational designs. An evidence base of what works for creating effective designs and what does not will facilitate designs that can improve access, individualization, and treatment engagement for consumers. This should allow for the creation of eHealth services that are more usable; engaging; and, therefore, effective.

### Conclusions

Seven of the key variables that influence how effective eHealth intervention designs are for those with and without mental health disorders are navigational depth, number of hyperlinks per page, presence of a pop-up menu, reading level, page length, the number of words per page, and a participant’s skills with the technologies.

### Future Research

Future work would benefit from a larger sample to better understand how these variables might interact with each other. This could allow the identification of specific ways in which the design environment interacts with design variables and synergistic or antagonistic effects of variable combinations. Each variable was studied at 2 levels only, which is common in a factorial design. Additional research could narrow the range of what is optimal. For example, a navigational depth of ≤3 was far superior to ≥5 levels, but the difference between 1, 2, and 3 levels might be considerable. However, it was not determined by these data. In addition, examination of variables other than these 12 variables would be very useful. A missing piece of this type of quantitative design research is user preferences for alternative designs. Coupling performance with preferences further advances the understanding of what works best for whom. We found that our single self-rated computer understanding question was very effective at measuring how savvy users were with the technologies. We are preparing a manuscript that fully describes this instrument and our findings. In addition, we are preparing a manuscript of our findings about the influence of the neurocognitive functions we collected from each participant on the effectiveness of the various designs and importance of the different design variables.

## Abbreviations

CART: classification and regression trees
SMI: severe mental illness
SSD: schizophrenia spectrum disorder
