# Correction: Key Variables for Effective eHealth Designs for Individuals With and Without Mental Health Disorders: 2^12-4 Fractional Factorial Experiment

**DOI:** 10.2196/31553

**Published:** 2021-07-07

**Authors:** Armando J Rotondi, Jonathan Grady, Barbara H Hanusa, Gretchen L Haas, Michael R Spring, Kaleab Z Abebe, James Luther, John Gurklis

**Affiliations:** 1 Mental Illness Research, Education and Clinical Center (MIRECC) VA Pittsburgh Health Care System Department of Veterans Affairs Pittsburgh, PA United States; 2 Center for Health Equity Research and Promotion (CHERP) VA Pittsburgh Health Care System Department of Veterans Affairs Pittsburgh, PA United States; 3 University of Pittsburgh Center for Behavioral Health, Media, and Technology University of Pittsburgh Pittsburgh, PA United States; 4 Computer Science Thomas College Waterville, ME United States; 5 Department of Psychiatry School of Medicine University of Pittsburgh Pittsburgh, PA United States; 6 Department of Information Sciences and Technology School of Information Sciences University of Pittsburgh Pittsburgh, PA United States; 7 Center for Research on Health Care Data Center School of Medicine University of Pittsburgh Pittsburgh, PA United States; 8 Behavioral Health VA Pittsburgh Health Care System Department of Veterans Affairs Pittsburgh, PA United States

In “Key Variables for Effective eHealth Designs for Individuals With and Without Mental Health Disorders: 2^12-4 Fractional Factorial Experiment” (J Med Internet Res 2021;23(3):e23137), two display errors were noted.

- In Table 1, row “Screen length,” column “Levels of dimensions in the factorial design; High,” the character “>” was not rendered in the originally published version. The value “1” has been corrected to “>1”.
- In Table 1, row “Constant navigational toolbar,” column “Levels of dimensions in the factorial design; High,” the value was not rendered in the originally published version. This value has been corrected to “1”.

In addition, five corrections have been made to the wording in Table 6:

- In row "The best performing websites had these design elements," "Mental illness other than SSD;" column "Design elements;" the phrase "screen per page length ≤1" has been corrected to "page length ≤1 screen long."
- In row "The best performing websites had these design elements," "No mental illness;" column "Design elements;" the phrase "screen per page length ≤1" has been corrected to "page length ≤1 screen long."
- In row "The worst performing websites with low navigational depth (≤3 levels) had these design elements," "Mental illness other than SSD;" column "Design elements;" the phrase "Screen per page length >1" has been corrected to "Page length >1 screen long."
- In row "Variables that, when present, always had a positive effect on performance," the subrow "Screen per page length ≤1" has been corrected to "Page length ≤1 screen long."
- In row "Variables that, when present, always had a negative effect on performance," the subrow "Screen length >1 screen long" has been corrected to "Page length >1 screen long."

The correction will appear in the online version of the paper on the JMIR Publications website on July 7, 2021, together with the publication of this correction notice. Because this was made after submission to PubMed, PubMed Central, and other full-text repositories, the corrected article has also been resubmitted to those repositories.

